# Correction: ‘Vampire bats rapidly fuel running with essential or non-essential amino acids from a blood meal’ (2024), by Rossi and Welch

**DOI:** 10.1098/rsbl.2025.0088

**Published:** 2025-08-06

**Authors:** Giulia S. Rossi, Kenneth C. Welch

**Affiliations:** ^1^ Department of Biological Sciences, University of Toronto Scarborough, Toronto, Ontario M1C 1A4, Canada; ^2^ Department of Biology, Hamilton, Stockholm, ON 101 33, Sweden

*Biol. Lett.* 20, 20240453 (Published online 06 November 2024). (https://doi.org/10.1098/rsbl.2024.0453)

Following the publication of our study entitled, ‘Vampire bats rapidly fuel running with essential or non-essential amino acids from a blood meal’, published in *Biology Letters* in 2024 [1] an astute reader alerted us to an error in the data we included. Specifically, the reader noted that the cost of transport (CoT; J m^−1^) data we presented did not seem plausible and asked us for clarification. Upon re-examination of the spreadsheet summarizing the data analysed in our article, we discovered that these data were not CoT data at all, but were a miscopied column of data that incorrectly calculated metabolic rate in W. Subsequently, we have recalculated both total (gross) and net CoT data. We show the corrected figure 2 below, specifically replacing the errant panel (*D*) with correct total and net CoT data. We have also corrected text in the Material and methods section of the manuscript to specify that both total and net CoT were calculated. We have revised three sentences in the Results section to report the corrected total and net CoT data. We have also amended the Discussion section to include a short reflection on the CoT data and its interpretation but conclude that the lack of more data on the cost of locomotion within each of the two observed gaits frustrates further interpretation of CoT data. Textual corrections to the article and online electronic supplementary material are highlighted in the accompanying electronic supplementary material.

**Figure 2.**
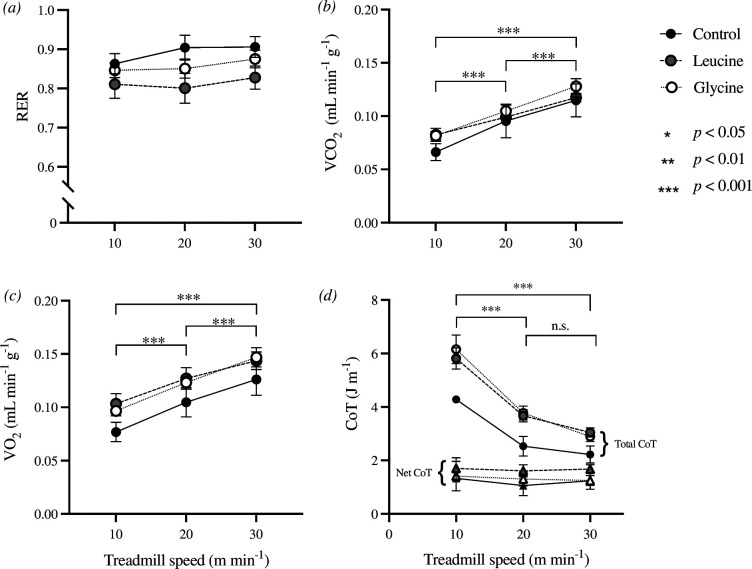
(*a*) The average respiratory exchange ratio (RER), (*b*) rate of carbon dioxide production (VCO_2_), (*c*) rate of oxygen consumption (VO_2_) and *d*) total and net cost of transport (CoT) in *Desmodus rotundus* at 10, 20 and 30 m min^-1^. Asterisks denote significant differences between treadmill speeds when a significant main effect of ‘speed’ was detected (mixed effect model, *p*<0.05). Net CoT (triangle symbols) did not differ significantly among speeds. Error bars represent the standard error.

Importantly, the erroneous CoT data we originally reported were not key data relative to our focal hypotheses. Thus, this correction has a negligible effect on our conclusions and in no way alters the principle finding that these bats rapidly and extensively support their walking and running with newly ingested amino acids. We apologize for this error and again thank the reader that alerted us to this mistake.

## Data Availability

The electronic supplementary material is available online [2].

## References

[1] Rossi GS, Welch KC. 2024 Vampire bats rapidly fuel running with essential or non-essential amino acids from a blood meal. Biol. Lett. **20**, 20240453. (10.1098/rsbl.2024.0453) 39500370 PMC11537760

[2] Rossi GS, Welch KC. 2025 Supplementary material from: Correction: Vampire bats rapidly fuel running with essential or non-essential amino acids from a blood meal. Figshare. (10.6084/m9.figshare.c.7942495) PMC11537760 39500370

